# Secretome analysis of in vitro aged human mesenchymal stem cells reveals IGFBP7 as a putative factor for promoting osteogenesis

**DOI:** 10.1038/s41598-018-22855-z

**Published:** 2018-03-15

**Authors:** Arantza Infante, Clara I. Rodríguez

**Affiliations:** Stem Cells and Cell Therapy Laboratory, BioCruces Health Research Institute, Cruces University Hospital, Barakaldo, 48903 Spain

## Abstract

Aging is a complex biological process, which involves multiple mechanisms with different levels of regulation. Senescent cells are known to secrete senescence-associated proteins, which exert negative influences on surrounding cells. Mesenchymal stem cells (MSCs), the common progenitors for bone, cartilage and adipose tissue (which are especially affected tissues in aging), are known to secrete a broad spectrum of biologically active proteins with both paracrine and autocrine functions in many biological processes. In this report, we have studied the secreted factors (secretome) from human MSCs (hMSCs) and hMSCs-derived adipocytes which were induced to accumulate prelamin A, the immature form of the nuclear lamina protein called Lamin A, known to induce premature aging syndromes in humans and in murine models. Proteomic analysis from two different techniques, antibody arrays and LS-MS, showed that prelamin A accumulation in hMSCs promotes the differential secretion of factors previously identified as secreted by hMSCs undergoing osteogenesis. Moreover, this secretome was able to modulate osteogenesis of normal hMSCs *in vitro*. Finally, we found that one of the overexpressed secreted factors of this human aging *in vitro* stem cell model, IGFBP-7, is an osteogenic factor, essential for the viability of hMSCs during osteogenesis.

## Introduction

The aging process results in a loss of tissue homeostasis, driving a progressive deterioration of cellular functions mainly due to cellular damages accumulated throughout life^1^. This age related cell damage leads to stem cell exhaustion and altered intercellular communication, which are proposed to be the “integrative” hallmarks of aging and responsible for the functional tissue decline associated with aging^2^. MSCs secrete a myriad of factors, known as the “secretome” which have been shown to modulate several processes, such as cell proliferation and differentiation^3^. In this report, we propose the hypothesis that aging alters the composition of the hMSCs secretome, with functional consequences in the surrounding cells. To elucidate this matter we have taken advantage of our previously validated experimental model of human aging, based on the pharmacological induction of prelamin A accumulation (the unprocessed form of the nuclear lamina protein named Lamin A) in hMSCs by the use of the HIV protease inhibitor Tipranavir (TPV)^4,5^. Lamin A, encoded by the *LMNA* gene, is synthesized as a precursor protein, prelamin A, which undergoes a series of posttranslational modifications in its carboxy-terminal CAAX motif, including farnesylation and proteolytic processing, to yield Lamin A^6^. This finely regulated post-translational process can be disrupted (due to gene mutations or by pharmacological treatments) resulting in pathological accumulation at the nuclear envelope of immature forms of Lamin A, such as progerin (a truncated fom of prelamin A) and prelamin A, which are toxic for cells^7–9^. The use of TPV treatment inhibits the activity of ZMPSTE24, a zinc metalloproteinase which cleaves the farnesylated prelamin A to produce mature Lamin A^9^. As a consequence of TPV inhibition, farnesylated prelamin A accumulates in the nucleus of the cells. Accumulation of immature forms of Lamin A is the hallmark of a devastating group of the so-called laminopathies characterized by premature aging phenotypes, such as Hutchinson-Gilford progeria syndrome (HGPS), or mandibuloacral dysplasia (MADA), syndromes associated with severe effects in mesenchyme-derived tissues, such as bone, fat and cartilage^10,11^. Remarkably, prelamin A accumulation has been detected in normal aging cells^12–14^, thus, reinforcing its role in normal chronological aging as well. In order to gain a deeper understanding of the complex aging process, we have focused on the secretome of aged hMSCs and the potential repercussions of altered protein expression to neighboring cells. To this purpose, given the proven and critical paracrine functionality of the mesenchymal stem cells’s secretome^15^, we have taken advantage of a validated experimental human aging model based on hMSCs which accumulate prelamin A. This aging *in vitro* model recapitulates the phenotypes observed in patients and mouse models^4,5^ as well as hallmarks of aging^2^. Futhermore, this experimental human model has been essential to elucidate some of the molecular mechanisms governing the aging process^4,5^.

In order to identify dysregulated secreted factors caused by prelamin A accumulation which could be mediating altered paracrine signaling in aging hMSCs, we used two complementary proteomic approaches, antibody arrays and liquid chromatography-mass spectrometry (LC-MS). The secretomes from hMSCs and hMSCs-derived adipocytes, both either accumulating prelamin A (preA-hMSCs, preA-adipocytes) or not (ctrl-hMSCs, ctrl-adipocytes) were analyzed. Notably, we found a high proportion of differentially secreted osteogenesis-related proteins in the secretome from preA-hMSCs. We showed that this secretome can increase osteogenic differentiation of normal hMSCs. Furthermore, this study revealed the essential role of a factor overexpressed in the secretome from preA-hMSCs, IGFBP7, in osteogenesis of hMSCs.

## Results

### Profiling the hMSCs secretome under conditions of prelamin A accumulation

**Figure 1:**
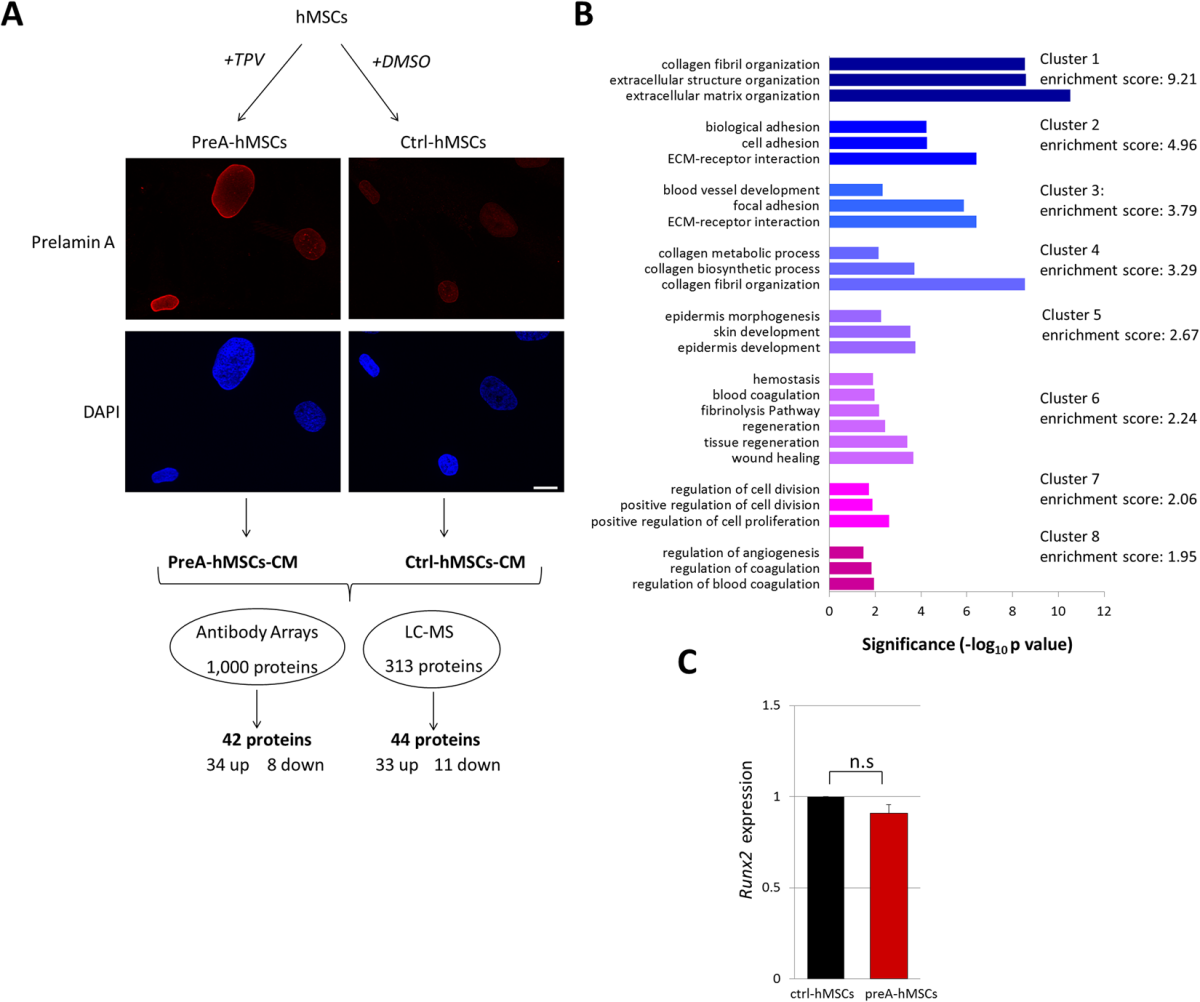
Analysis of preA-hMSCs-CM reveals altered secretion of proteins related to extracellular matrix, cell adhesion, angiogenesis and wound healing. (**A**) Schematic overview of hMSCs treatment to induce prelamin A accumulation. From each hMSCs line hMSCs accumulating prelamin A (preA-hMSCs) and control hMSCs (ctrl-hMSCs) were obtained in parallel. Prelamin A accumulation at the nuclear envelope was confirmed by confocal microscopy (red: prelamin A, blue: DAPI). Scale bar = 10 µm. Conditioned media from preA-hMSCs and ctrl-hMSCs were collected and subjected to proteomic analysis. Two independent hMSCs lines were use to obtain conditioned media in the case of antibody arrays (n = 2) and 4 independent hMSCs lines in the case of LC-MS (n = 4). (**B**) The differentially secreted proteins by preA-hMSCs (detected by antibody arrays and LC-MS) were interrogated in terms of functional annotation by DAVID Bioinformatics Resources. The representative Gene Ontology terms, grouped in clusters with an enrichment score of 1.5 or above are presented. The x-axis represents the significance (p value) for each term, while the y-axis represents the ontology categories. (**C**) Real-time quantitative PCR was used to assess the expression of *Runx2* in pre-hMSCs and ctrl-hMSCs. *Runx2* mRNA expression was normalized to the control gene *Gapdh* and fold induction was then calculated in reference to ctrl-hMSCs. Results are expressed as mean ± SEM (n = 4).

In order to identify the factors secreted by aged hMSCs, we took advantage of the prelamin A-accumulating mesenchymal stem cell model generated previously by our group^4,5^. Accumulation of prelamin A in hMSCs is induced by the presence of the HIV protease inhibitor Tipranavir (TPV), which also inhibits the activity of ZMPSTE24, essential for processing prelamin A to yield mature Lamin A^9^. In parallel, control hMSCs were incubated with the vehicle alone, dimethyl sulfoxide (DMSO) (Fig. 1A). This experimental model recapitulates many of the phenotypes of cell aging and has been fundamental to elucidate some of the molecular mechanisms underlying it^4,5^. Conditioned medium (CM) from preA-hMSCs (preA-hMSCs-CM) and from ctrl-hMSCs (ctrl-hMSCs-CM) were collected and compared first by semi-quantitative antibody arrays capable of detecting up to 1000 proteins of human serum, including cytokines, chemokines and growth factors (Fig. 1A). This analysis detected a dysregulation in the secretion levels of 42 proteins, most of them overexpressed, in preA-hMSCs-CM when compared to ctrl-hMSCs-CM, (Fig. 1A and Supplementary Table S1). The antibody arrays technique is considered to be a “targeted” technology, because the analysis is limited to the antibodies present in the array. To extend and complement this targeted analysis, we analyzed the preA-hMSCs-CM and ctrl-hMSCs-CM by LS-MS, an untargeted technique, theoretically capable of detecting any protein expressed in the secretome (Fig. 1A). LS-MS analysis identified the global secretion of more than 300 proteins in both ctrl-hMSCs-CM and preA-hMSCs-CM, and among these secreted proteins, 44 were detected as differentially secreted by preA-hMSCs, again the majority of them up-regulated (Fig. 1A and Supplementary Table S1). To determine the functional meaning of the dysregulated secreted proteins induced by prelamin A accumulation in hMSCs, we performed Gene Ontology analysis by integrating the results obtained by these two techniques (86 proteins differentially secreted), and found a significant over-representation of categories mainly related to extracellular matrix, specifically “collagen fibril organization”,“adhesion”, “angiogenesis” and “wound healing” (Fig. 1B). An in depth study of the literature, showed that, 54 of these dysregulated proteins (>60% of the differentially secreted proteins) were previously identified as differentially secreted by hMSCs undergoing osteogenesis^16,17^. Moreover, among these factors, we identified several proteins implicated in TGF-β signaling (Supplementary Table S1), a cytokine with a fundamental role in bone remodeling^18^.

The above findings led us to consider the possibility that prelamin A accumulation could be inducing a spontaneous osteoblastic differentiation of hMSCs, which could account for a secretome rich in osteogesis-related proteins. To test this possibility, and because it is an essential transcription factor for the commitment of hMSCs to an osteoblast lineage^19^, we examined *Runx2* gene expression in these cells, but found no differences in *Runx2* expression between ctrl-hMSCs and preA-hMSCs (Fig. 1C).

### Functional consequences of secretome from preA-hMSCs

**Figure 2:**
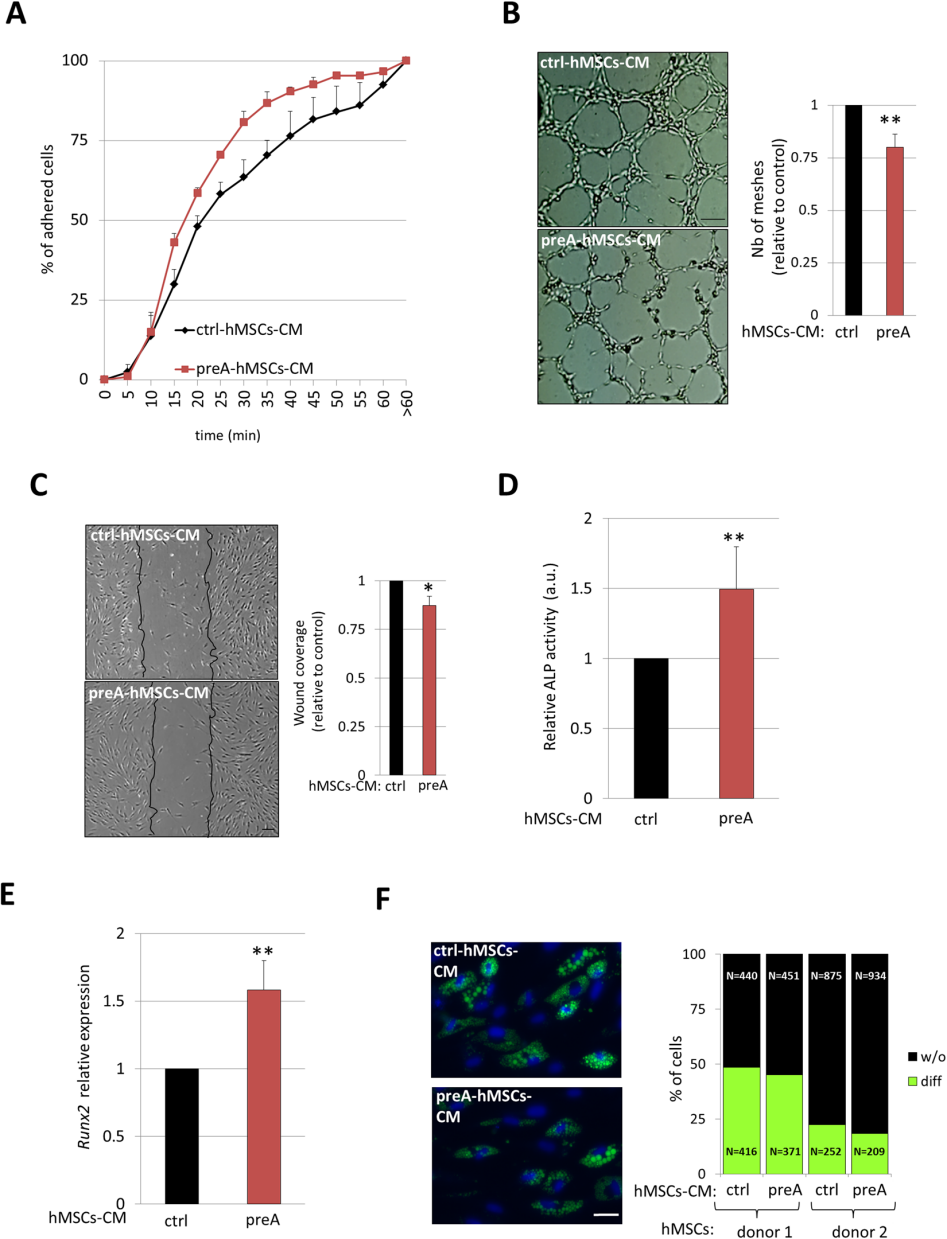
Functional analysis of preA-hMSCs-CM. (**A**) Normal hMSCs cultured in preA-hMSCs-CM attach to cell culture plates faster than hMSC under ctrl-hMSCs-CM. Results represent mean ± SEM (n = 3). (**B**) I*n vitro* tube formation assay of HUVECs cultured under ctrl o preA-hMSCs-CM for 4 hours. Representative bright field images are shown, scale bar = 100 µM. The graph presented on the right represents the mean ± SD of the number of meshes quantified by ImageJ software (n = 5). **(C)** *In vitro* scratch assay showing the wound coverage of normal hMSCs after 24 hours of cell culture in the presence of ctrl or preA-hMSCs-CM. Representative bright field images show black lines indicating the wound borders at the beginning of the assay. Scale bar = 100 µM. The graph presented on the right represents the mean ± SD of wound coverage (n = 4). 6 days after osteogenic differentiation of normal hMSCs under the presence of preA or ctrl-hMSCs-CM, ALP activity **(D)** and *Runx2* expression **(E)** were assessed. Results are expressed in reference to data obtained from hMSCs cultured under ctrl-hMSCs-CM and represent mean ± SD (n = 6). *Runx2* mRNA expression was normalized to the control gene *Gapdh*. **(F)** Representative microscopy images of hMSCs-derived adipocytes, after 21 days of adipogenesis under the presence of preA or ctrl-hMSCs-CM. The percentage of differentiated adipocytes was determined by counting the number of cells containing lipid droplets stained with Bodipy (green). The total number of cells was counted by DAPI nuclei staining (blue). Scale bar = 20 µm. The graph on the right represents the percentage of differentiated adipocytes (diff) and not differentiated hMSCs (w/o), and it was determined in 2 independent hMSCs lines (n = 2).

Subsequently, we functionally challenged the preA-hMSCs-CM in normal cells. Considering the *in silico* analysis outcome, we first focused on cell adhesion, angiogenesis and wound healing, processes found to be significantly enriched by the GO analysis of the dys-regulated factors secreted by preA-hMSCs. Consistent with a secretome rich in adhesion proteins, preA-hMSCs-CM was found to be pro-adhesive. Thus, normal hMSCs attached to cell culture plates in an accelerated fashion when cultured under preA-hMSCs-CM compared to normal hMSCs under ctrl-hMSCs-CM (Fig. 2A). In contrast, preA-hMSCs-CM slightly repressed both wound healing in normal hMSCs (Fig. 2C) and angiogenesis in HUVECs (Fig. 2B,C).

It has been previously described that human osteoblast–derived factors induce early osteogenic markers in hMSCs^20^. We therefore tested whether the secretome from preA-hMSCs, rich in osteogenesis-related secreted factors, could induce paracrine osteogenic signals in hMSCs. In order to study this posibility, we challenged normal hMSCs to differentiate in osteogenesis induction medium (OIM) for 6 days in the presence of preA-hMSCs-CM or ctrl-hMSCs-CM. We then analyzed *Runx2* expression and alkaline phosphatase (ALP) activity in these cells, both established markers of early osteoblasts differentiation. Interestingly, at this initial stage of differentiation, hMSCs cultured under CM from preA-hMSCs presented increased *Runx2* gene expression and ALP activity when compared with hMSCs cultured under ctrl-hMSCs-CM (Fig. 2D,E), supporting the osteogenic potential of preA-hMSCs-CM.

Since hMSCs commitment towards osteoblast lineage accompanies an inhibition of adipocyte lineage, we wondered whether prelamin A secretome from hMSCs could affect adipocyte differentiation in hMSCs. We induced adipogenesis in hMSCs for 21 days, in the presence of preA-hMSCs-CM or ctrl-hMSCs-CM. At the end of the differentiation period, the percentage of adipogenic differentiation in the cell culture was assessed by examining lipid droplet accumulation inside the cells. Adipocytes were distinguished from undifferentiated hMSCs by Bodipy fluorescent labeling, which stains the neutral lipids stored inside lipid droplets of adipocytes. Notably, preA-hMSCs-CM had no significant effect on adipogenesis of hMSCs (Fig. 2F), suggesting that the factors present in the prelamin A secretome from hMSCs induces the activation of osteoblastic differentiation pathways, but have little effect on adipogenic differentiation.

### Prelamin A accumulation specifically regulates the altered secretion of extracellular matrix proteins in both hMSCs and hMSCs-derived adipocytes

**Figure 3:**
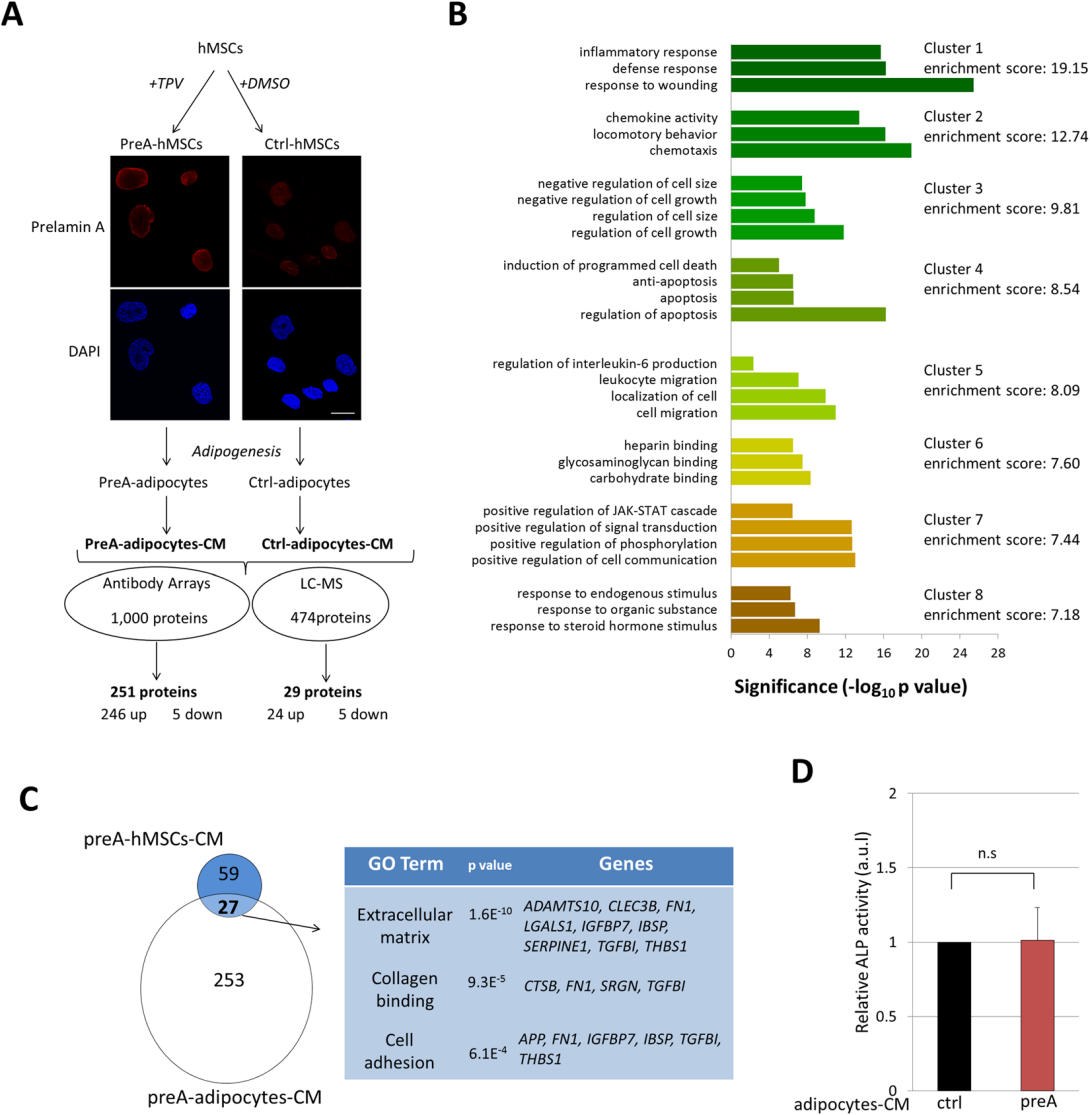
Secretome analysis of preA-adipocytes differentiated from hMSCs. (**A**) Schematic overview of hMSCs cell culture, induction of prelamin A accumulation by TPV treatment, adipogenesis, obtaining CM from hMSCs-derived adipocytes, and subsequent secretome analysis by antibody arrays and LC-MS approaches. Before adipogenic differentiation, induction of prelamin A accumulation in hMSCs was confirmed by confocal microscopy, red: prelamin A, blue: DAPI. Scale bar = 10 µm. **(B)** Functional annotation clustering of differentially secreted proteins in CM from preA-adipocytes, determined using the DAVID bioinformatic tool. The representative GO terms, grouped in clusters with an enrichment score of 7 or above are presented. The x-axis represents the significance (p value) for each term, while the y-axis represents the ontology categories. **(C)** Venn diagrams showing overlap of 27 proteins between differentially secreted proteins by preA-hMSCs and preA-adipocytes. Gene ontology analysis of these proteins revealed significant over-representation of categories related to extracellular matrix, collagen binding and cell adhesion. (**D**) Six days after osteogenic differentiation of normal hMSCs in the presence of preA-adipocytes-CM or ctrl-adipocytes-CM, ALP activity was assessed. Results are expressed in reference to ALP activity of hMSCs cultured under ctrl-adipocytes-CM and represent mean ± SD, n = 6.

To explore the possibility that prelamin A accumulation could be mediating the secretion of specific proteins in different cell types of mesenchymal origin, we differentiated our experimental model to induce adipocyte formation from hMSCs accumulating prelamin A (preA-adipocytes)^4^ (Fig. 3A). We demonstrated in previous works that these preA-adipocytes exhibit an age-related lipid metabolic profile, as well as characteristics of premature aging^4,21^.

The conditioned medium (CM) from preA-adipocytes (preA-adipocytes-CM) and ctrl-adipocytes (ctrl-adipocytes-CM) were collected and compared by semi-quantitative antibody arrays and LC-MS. We found that CM from preA-adipocytes was more complex than pre-hMSCs-CM in terms of the total number of dysregulated secreted proteins. The antibody arrays technique identified more than 250 proteins which were differentially secreted under prelamin A accumulation in hMSCs-derived adipocytes (Fig. 3A and Supplementary Table S2). Using LC-MS more than 400 secreted proteins in both preA-adipocytes-CM and ctrl-adipocytes-CM were detected, among them 29 proteins being differentially secreted by preA-adipocytes. Integrated GO analysis from both sets of dysregulated factors (280 secreted proteins total) showed significant enrichment in categories related to inflammatory response, chemokine activity and cell growth (Fig. 3B).

To search for the intersection of possibly overlapping dysregulated secreted proteins in both preA-hMSCs and preA-adipocytes, we next compared the differentially secreted factors by both types of cells. Interestingly, this comparison yielded 27 proteins commonly differentially secreted by both preA-hMSC and preA-adipocytes (Venn diagram, Fig. 3C). This common set of proteins were significantly grouped in categories related to extracellular matrix, collagen binding and cell adhesion through Gene Ontology analysis (Fig. 3D). These results suggest that prelamin A accumulation specifically induces the dysregulation of extracellular matrix proteins in hMSCs and in hMSC-derived adipocytes. Contrary to the results observed in preA-hMSCs-CM, a small proportion of dysregulated secreted proteins were described as osteogenesis-related factors (41 of these 280 proteins; <15% of the total differentially secreted proteins)^16,17^, and 28 of these 41 proteins matched the osteogenesis-related secreted proteins in preA-hMSCs-CM (Supplementary Table S1). Consistent with this low proportion of dysregulated osteogeneic-related factors, the secretome from preA-adipocytes did not induce an increase of ALP activity in hMSCs when compared with the CM from ctrl-adipocytes (Fig. 3D).

Taken together, these results show that prelamin A accumulation specifically induces the dysregulation of extracellular matrix proteins in both hMSCs and in hMSC-derived adipocytes, but the number of affected secreted factors and the level of their dysregulation is quite different between the preA-adipocytes and the preA-hMSC.

### IGFBP7 expression is essential for maintaining hMSCs viability during osteogenesis

**Figure 4:**
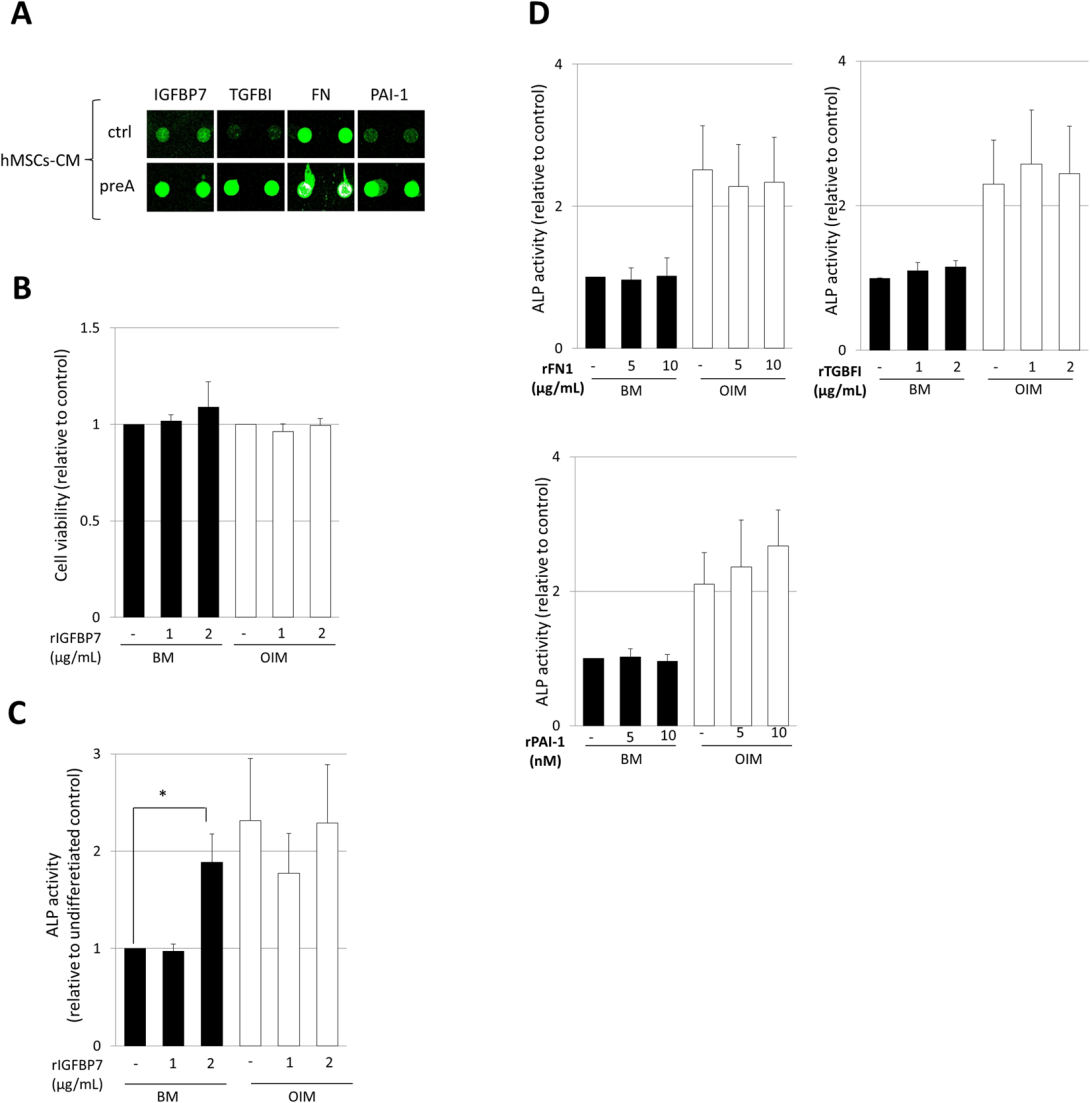
IGFBP7 is identified as a osteogenic inductor in normal hMSCs. (**A**) Fluorescence images of antibody arrays obtained via laser scanning, showing spot signal intensities for TGFβ target genes, IGFBP7, TGFBI, FN and PAI-1. Duplicated spots for each protein are shown in the image. (**B**) Cell viability assay performed by CCK-8 kit at day 6 of cell culture under basal medium (BM) or osteogenesis induction medium (OIM). Results are normalized to those obtained from hMSCs without rIGFBP7 treatment under both BM or OIM cell culture conditions (n = 4). **(C**,**D)** ALP activity analysis in normal hMSCs after incubation with rIGFBP7, rFN, rTGFBI or rPAI-1 in BM or OIM during 6 days. Results are normalized to those obtained from hMSCs without rIGFBP7 treatment under BM (n = 4) (*p < 0.05, n = 4).

Among the identified secreted factors in the proteomic analysis, a set of secreted factors which are known TGF-β targets drew our attention. Some of these TGF-β targets, FN1, TGFBI, IGFBP7 and PAI-1 were significantly up-regulated (≥ten-fold of induction) in preA-hMSCs-CM (Fig. 4A) (Supplementary Table S1), suggesting a possible role for these proteins in the increased osteogenesis observed in hMSCs cultured using this altered CM. To explore this possibility, we incubated normal hMSCs with recombinant human FN1, TGFBI, IGFBP7 and SERPINE1 under basal medium (BM) or osteogenesis induction medium (OIM) for 6 days, similar to the time frame in which the preA-hMSCs-CM exhibited their osteogenic potential. Remarkably, only rIGFBP7, which did not alter hMSCs viability (assessed by Cell Counting Kit-8; CCK-8) (Fig. 4B), could induce an increase in ALP activity in hMSCs using basal medium, (Fig. 4C,D), suggesting that IGFBP7 alone in the cell culture medium can induce the early steps of osteogenesis in hMSCs without the addition of any additional factors.

**Figure 5:**
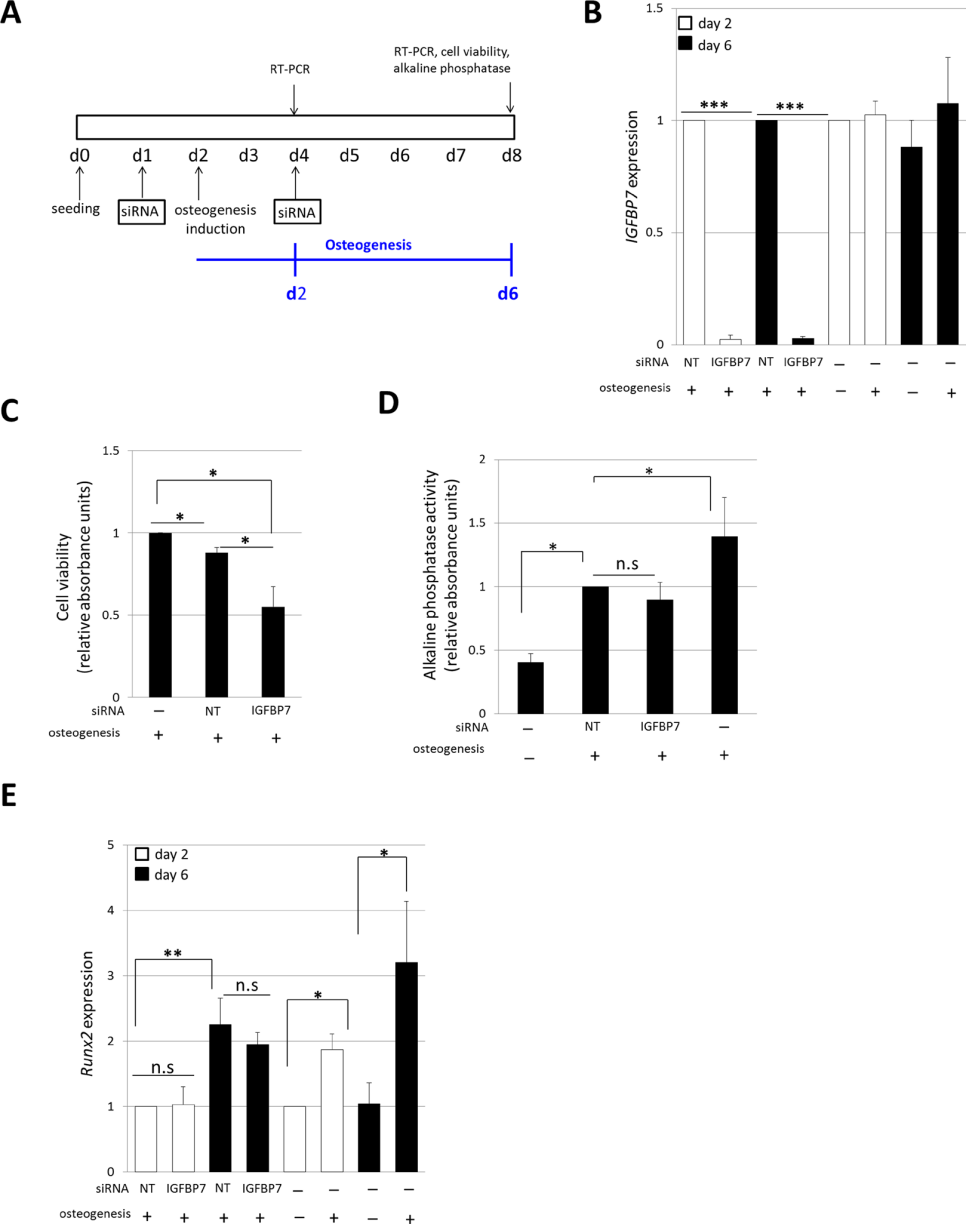
IGFBP7 expression is essential for hMSCs survival during early osteogenesis. (**A**) Scheme of the workflow carried out to study the effects of *IGFBP7* silencing during hMSCs early osteogenesis. Two rounds of siRNA were performed to ensure the continuous silencing of *IGFBP7* during the 6 days (d) of osteogenesis differentiation. The effects of *IGFBP7* silencing were assessed after 2 and 6 days of osteogenesis of hMSCs. (**B**) *IGFBP7* mRNA expression was studied by RT-PCR after 2 and 6 days of osteogenesis induction. *IGFBP7* expression in siIGFBP7 hMSCs was normalized to *Gapdh* and fold induction was calculated in reference to hMSCs transfected with siNT (n = 5). In parallel, the expression of *IGFBP7* was studied in non-transfected hMSCs. *IGFBP7* fold induction was calculated in reference to hMSCs without differentiation induction at day 2 of cell culture (n = 5). (**C**) Cell viability after *IGFBP7* silencing was assessed after 6 days of osteogenic differentiation. Transfected hMSCs viability was normalized to that observed in non-transfected hMSCs (n = 5). (**D**) Alkaline phosphatase activity was assessed at day 6 of osteogenesis after siRNA. Results are normalized to the alkaline phosphatase activity of hMSCs transfected with siNT (n = 5). Negative and positive controls of osteogenesis, without transfecting are shown. (**E**) *Runx2* expression was assessed at day 2 and 6 of osteogenesis after siRNA and was normalized to *Gapdh. Runx2* fold induction was calculated in reference to *Runx2* expression of hMSCs transfected with siNT at day 2. As a control, the expression of *Runx2* was also studied in non-transfected hMSCs at day 2 and 6 of cell culture. In this case, *Runx2* fold induction is calculated in reference to *Runx2* expression in hMSCs without differentiation at day 2 of cell culture (n = 5). Results are expressed as mean ± SD, *** indicates p < 0.001; ** indicates p < 0.01 and * indicates p < 0.05.

To study a possible role for IGFBP7 in hMSCs osteogenesis, we silenced IGFBP7 expression during early osteogenesis of hMSCs (6 days of differentiation) using an RNA interference approach. Thus, hMSCs were transfected with a siRNA targeting *IGFBP7* (siIGFBP7) or a control, non-targeting siRNA (siNT) prior to initiation of differentiation as well as during the differentiation process (Fig. 5A). mRNA levels of *IGFBP7* were confirmed to be significantly reduced at day 2 and day 6 of osteogenic differentiation by 90% (p < 0.001, n = 5) when compared to control cells transfected with siNT (Fig. 5B). We then evaluated the effects of *IGFBP7* silencing on hMSCs viability during osteogenic differentiation by CCK-8 assay. The results showed that hMSCs viability during early osteogenic differentiation was notably reduced (≈50%) in *IGFBP7* siRNA transfected cells when compared with control siRNA transfected cells (Fig. 5C). On the other hand, the silencing of *IGFBP7* hampered neither alkaline phosphatase activity, nor *Runx2* expression, in hMSCs (Fig. 5D,E) during early osteogenic differentiation (Fig. 5D,E).

Taken together, these results suggest that during early osteogenic differentiation, IGFBP7 is essential for hMSCs viability, but not for the expression of early osteogenesis markers.

## Discussion

In this study, we have analyzed the secreted proteins associated with prelamin A-induced aging in hMSCs and hMSCs-derived adipocytes by integrating the data from two complementary proteomic methods: a targeted, semi-quantitative analysis, based on human antibody arrays and an unbiased quantitative analysis by LC-MS proteome identification. The antibody arrays technique is a high throughput technology for detecting protein expression with high sensivity, and it can detect proteins expressed at very low levels, such as cytokines, growth factors, etc. However, it is a “targeted” technology since only proteins present in the array can be detected. On the contrary, unbiased technologies such as mass spectrometry try to separate and quantify every protein of the expressed proteome, but in this case, low abundant proteins are masked by high abundance proteins^22^. In our study, only 2 proteins in the case of preA-hMSCs-CM (pentraxin and calreticulin) (Supplementary Table S1), and none in preA-adipocytes-CM (Supplementary Table 1) were overexpressed in both techniques, as expected considering the complementary, non overlapping features of these proteomic approaches.

Altered secreted proteins due to prelamin A accumulation were analyzed in terms of Gene Ontology and an over-representation of proteins related mainly to extracelular matrix, from both hMSCs and hMSCs-derived adipocytes was observed. This result reinforces previous data from our group^4,5^ and others^23,24^ regarding dysregulation of extracellular matrix genes at the transcriptome level under prelamin A accumulation in hMSCs. Taken together, these data show that the effects of prelamin A accumulation in extracellular matrix dysregulation have consequences at multiple levels of cell regulation, such as transcription and protein secretion.

It is well known that human aging is associated with a progressive decline in bone mass^25^, but surprisingly we found that the secretome from aged hMSCs, is highly enriched in osteogenesis-related secreted proteins^16,17^. Consequently, this secretome from preA-hMSCs stimulates the first stages of osteogenesis in normal hMSCs, but has no evident effect in hMSCs adipogenesis. These results demonstrated that differentially secreted factors by hMSCs due to prelamin A accumulation can influence commitment of normal hMSCs in differentiation, specifically osteogenesis, via paracrine signaling. Conversely, CM from preA-adipocytes did not show differences in osteogenic activity when compared with CM from ctrl-adipocytes, in spite of having among its factors more than 40 proteins known to be secreted in hMSCs osteogenesis. Posible explanations for this result could be the differences in the fold of induction of the osteo-related dysregulated factors (considerably lower in preA-adipocytes), and/or the presence of other factors in the preA-adipocytes secretome that could counterbalance a possible osteogenic induction.

The osteogenic features of the secretome from preA-hMSCs, suggested that prelamin A accumulation in hMSCs could be inducing a spontaneous differentiation of hMSCs to osteoblastic lineage under basal cell culture conditions. Indeed, previous studies in premature aging cell models have shown that the expression of progerin in inmortalized hMSCs^23^ and in hMSCs derived from HGPS patients^26–28^, induces an increased osteogenesis in these cells upon osteogenesis induction reflecting a “premature” osteogenesis. We did not detect any increase in the expression of *Runx2* in preA-hMSCs, in the present study, nor was *Runx2* upregulation detected in previous studies performed with cDNA microarrays^5^, but we can not rule out the possibility that under osteogenesis induction, prelamin A accumulation could enhance osteogenic differentiation of hMSCs.

Among the dysregulated secreted factors by preA-hMSCs, four known TGF-β targets were highly up-regulated (≥ten-fold of induction): FN1, TGFBI, IGFBP7 and SERPINE1. TGF-β signaling is known to play a crucial role in osteogenesis, affecting hMSCs fate and therefore bone remodeling^18^. Indeed, excessive TGF-β signaling has been proposed as a mechanism involved in bone pathologies such as osteogenesis imperfecta^29^ and osteoporosis^30^. Interestingly, Lamin A has been previously described as a modulator of downstream effects of TGF-β1 signaling^31^ and our findings also suggested prelamin A accumulation affects regulation of TGF-β activity. Consistent with this observation, it has been reported that osteoblast-like cells and serum samples from MADA patients (which accumulate prelamin A) show an increase in TGFβ−2 secretion^32,33^.These factors showed upregulation in the secretome from preA-adipocytes as well, although the fold of induction was much lower in most cases.

Of the four factors clearly overexpressed in preA-hMSCs, only IGFBP7 induced an increase in alkaline phosphatase activity in hMSCs. IGFBP7, a member of the IGFBP superfamily, is a secreted protein binding insulin, activina A and type IV collagen among others^34^, and its expression is known to be regulated by TGF-β1^35^. It has been described to exert its functions in biological processes such as cellular proliferation, adhesion and angiogenesis^34^. It was also identified as a factor secreted by senescent hMSCs^36^, which in turn, induces senescence in younger hMSCs at high concentrations (10 µg/mL) of the recombinant protein. To avoid a possible induction of senescence in hMSCs by IGFBP7, we used lower concentrations of rIGFBP7 in this study, finding that it was capable of inducing an increase in alkaline phosphatase activity, suggesting an increase in early osteogenesis in hMSCs cultured under basal medium but not under osteogenesis induction medium. This result suggested that at the assayed concentrations, IGFBP7 *per se* could activate osteogenesis pathways, but did not enhance the signaling pathways activated by OIM factors (dexamethasone, ascorbic acid, β-glycerophosphate). In agreement with this observation, a recent work indicated that IGFBP7 enhanced the osteogenesis in hMSCs cultured in OIM only when used at higher concentrations (10 µg/mL)^37^.

Intriguingly IGFBP7 silencing in hMSCs during the early osteogenesis process led to a significant decreased viability of cells (≈ 50%) and no effect in the expression of osteoblastic markers in remaining cells (ALP activity and *Runx2* expression). Given the fact that in the first step of osteogenesis, the commitment stage, the hMSCs intensely proliferate by asymmetric cell division, giving rise to another stem cell and a commited osteoprogenitor^38^, we speculate that IGFBP7 expression could be crucial for hMSCs viability during the first steps of osteogenesis. Further studies will clarify if this diminished cell viability due to IGFBP7 knockdown is due to a slower cell division or an increase apoptosis of hMSCS.

Taken together, our results surprisingly show that the secretome from aged hMSCs (due to prelamin A accumulation) leads to an aberrant paracrine signaling which triggers an accelerated early osteogenesis. Importantly, this is consistent with a previous work focused on vascular aging, which showed that ectopic expression of prelamin A in vascular smooth muscle cells (VSMCs) caused the induction of cell senescence and the secretion of paracrine factors which in turn, induced osteogenesis (measured in terms of ALP activity) in mesenchymal precursors^14^. This work showed that prelamin A accumulated in calcifying VSMCs, often seen in elderly people, both *in vitro* and *in vivo*. Taken together, these results highlight the idea that this “early” accelerated osteogenesis is a part of the aging process of bone, contributing to an unbalanced homeostasis of bone tissue. Thus, HGPS patients, whose cells show increased *in vitro* osteogenesis, exhibit high bone-turnover and severe skeletal dysplasia with severe abnormalities in bone growth and morphology^39^. In line with this concept, a recent publication of experiments performed in mice showed that pre-treatment of whole-mouse bone marrow with conditioned medium from senescent cells led to an enhanced osteoclast differentiation potential of these cells^40^, which is associated with higher bone resorption and consequently, increased bone loss.

Overall, the present work demonstrates that aged hMSCs, the progenitors of bone cells, can modulate osteogenesis by means of paracrine signaling via a secretome rich in osteogenesis-related proteins. Moreover, we have found that one of the differentially secreted factors induced by prelamin A accumulation, IGFBP7, has a fundamental role in maintaining viability of hMSCs during the commitment stage of osteogenesis. Since bone is constantly renewed by the balanced action of osteoblastic bone formation and osteoclastic bone resorption, further studies focusing on other processes participating in bone homeostasis such as mineralization and osteoclastogenesis will be crucial to determine the mechanisms of pathological paracrine signaling in aged cells.

## Methods

### hMSCs cell culture and prelamin A accumulation induction

Bone marrow hMSCs derived from healthy donors were commercially obtained (Lonza, Cat N°: PT-2501) and cultured in BM: DMEM low glucose with glutamax (Thermofisher, Cat N°: 11570586), FBS 10% (Sigma, Cat N°: F7524) and 1% penicillin/streptomycin (Thermofisher, Cat N°: 11548876). Induction of prelamin A accumulation and adipogenesis differentiation was performed in bone marrow derived hMSCs lines (Supplementary Table S3) as described previously^4^. Briefly, to induce prelamin A accumulation, passage 3–4 hMSCs were treated with the HIV protease inhibitor tipranavir (30 µM) every other day until passage 11. In parallel, ctrl-hMSCs were incubated with the vehicle, DMSO. For adipogenic differentiation, when hMSCs under TPV treatment reached passage 11 they were seeded at confluence, and the following day induced to differentiate by culturing them 21 days in the presence of adipogenic factors: 1 µM dexamethasone (Sigma, Cat N°: D4902), 500 µM 3-isobutyl-1-methylxantine (Sigma, Cat N°: I5879), and 200 µM indomethacin (Sigma, Cat N°: I7378). TPV treatment was maintained during the 21 days of differentiation.

For prelamin A accumulation detection, hMSCs or adipocytes derived from hMSCs were grown on glass coverslips and fixed in paraformaldehyde 4% (Sigma, Cat N°: HT501128). Immunostaning was carried out as described previously^41^ using a specific antibody recognizing prelamin A (Santa Cruz, Cat N°: sc-6214). DAPI (Sigma, Cat N°: D9542) was used to counterstain cell nuclei as described previously.

### Conditioned medium collection

To analyze the secretome, CM was recovered from cells cultured under FBS-free BM during the last 24 hours of culture. For functional experiments with CM, normal hMSCs (passages 6–8) were used.

Recombinant human IGFBP7, SerpinE1, Fibronectin and TGFBI (R&D Systems, Cat N°: 1334-B7, 1786-PI, 4305-FN and 3409-GB respectively) were independently added to cell culture at the indicated final concentrations and incubated for 6 days. Media and factors were replaced at day 3.

### Antibody Arrays

For antibody array experiments, pooled CM from 2 independent biological hMSCs (n = 2) and pooled CM from 2 independent hMSCs-derived adipocytes lines (n = 2) were used. The RayBio® Label-based (L-Series) Human Antibody Array 1000 kits (CatN°: AAH-BLG-1000) were used following the manufacturer’s instructions (RayBiotech). Scanned images were captured using an Axon GenePix laser scanner. Protein intensities were determined by taking the average of the 2 spots specific to each protein, and all protein intensities on a given array were normalized against the blank, negative control and positive control spots.

### Protein digestion and LC-MS analysis

LC-MS analysis was executed in the Proteomics Platform at CIC Biogune (Derio, Spain). For these studies, CM from 4 independent biological hMSCs and hMSCs-derived adipocytes lines (n = 4) were processed and analyzed independently. Samples were digested following the FASP protocol^42^ with minor variations. Peptides were cleaned with C18 Zip Tip stage tips (Millipore) and loaded onto a nanoACQUITY UPLC System (Waters) connected to a Synapt G2Si ESI Q-Mobility-TOF spectrometer (Waters) equipped with an ion mobility chamber (T-Wave-IMS) for high definition data acquisition analyses. An aliquot of each sample was loaded onto a Symmetry 300 C18 UPLC Trap column. The precolumn was connected to a BEH130 C18 column, and equilibrated in 5% acetonitrile and 0.1% FA. Peptides were eluted directly into the nanoelectrospray capillary (Proxeon Biosystems), using a chromatographic ramp of 120 min. A lock mass compound [Glu1]-Fibrinopeptide B (100fmol/ul) was delivered by an auxiliary pump of the LC system at 500 nl/min to the reference sprayer of the NanoLockSpray (Waters) source of the mass spectrometer. 0.5 ug of each sample were loaded for each run. Data were post-acquisition lock mass corrected using the double charged monoisotopic ion of [Glu1]-Fibrinopeptide B. Accurate mass LC-MS data were collected in HDDA mode which enhances signal intensities using the ion mobility separation step.

Progenesis LC-MS (version 4.0.4265.42984, Nonlinear Dynamics) was used for the label-free differential protein expression analysis. After importing the Raw files from the MS acquisition of the samples to the program, one of the runs was used as the reference to which the precursor masses in all other samples were aligned. Only features comprising charges of 2+ and 3+ were selected. The raw abundances of each feature were automatically normalized and logarithmized against the reference run. Samples were grouped in accordance to the comparison being performed, and an ANOVA analysis was performed. Features with an ANOVA p-value ≤ 0.05 and a ratio >2 in either direction were considered further. A peak list containing the information of these significantly different features was generated and exported to the Mascot search engine (Matrix Science Ltd.).

The generated.mgf file was searched against Uniprot/Swissprot human database, considering carbamidomethylation of cysteines as fixed modification and oxidation of methionines as variable modification. Database searching was performed using MASCOT 2.2.07 (Matrixscience, London, UK) against a UNIPROT - Swissprot database filled only with entries corresponding to Homo sapiens (without isoforms). For protein identification the following parameters were adopted: carbamidomethylation of cysteines (C) as fixed modification and oxidation of methionines (M) as variable modifications, 15 ppm of peptide mass tolerance, 0.2 Dalton fragment mass tolerance and up to 3 missed cleavage points, peptide charges of +2 and +3. Only hits with a FDR <1% were kept. The list of identified peptides was imported back to Progenesis LC-MS, and the previously quantified features were matched to the corresponding peptides. Non-conflicting peptides (peptides occurring in only one protein) were specifically chosen for quantitative purposes, and only proteins with at least two quantified non-conflicting peptides were selected. The significance of expression changes was again tested at the protein level, and proteins not satisfying the ANOVA p-value ≤ 0.05 and Ratio >2 in either direction criteria were filtered out.

### Functional annotation

For Gene Ontology (GO) enrichment analysis of Biological Processes, we used the Database for Annotation, Visualization, and Integrated Discovery^43^ (DAVID; http://david.abcc.ncbifcrf.gov) as we previously described^41^. Thus, the differentially dys-regulated proteins identified by both antibody arrays and LS-MS were loaded into DAVID and submitted to the functional annotation clustering tool. We only considered the enriched GO terms generated by modified a Fisher Exact test followed by the Bonferroni test and *p* value threshold of <0.05^43^.

### Live microscopy for cell adhesion, tube formation and angiogenesis

All live microscopy experiments were performed on a Nikon Eclipse TE 2000 microscope and the images were obtained with NIS software (Nikon).

To monitor cell adhesion under CM, 45,000 hMSCs resuspended in 600 µL of CM were seeded in 12-well plates. Immediately after seeding, a field in the center of the well was selected with at least 100 cells per condition and the first image was taken to set the zero time-point. Then, images were acquired every 5 minutes for a total imaging period of 2 hours. Adhered cells were counted for each time period. Three biological replicates were used to perform adhesion studies (n = 3) with CM from hMSCs coming from three independent donors.

For tube formation assays, 10 µL of Growth Factor Reduced Matrigel (Corning, Cat N°: 356230) (thawed previously overnight at 4 °C) were used to coat µ-Slide Angiogenesis wells (Ibidi, Cat N°: 81501), to form a layer of gel matrix of 0.8 mm thick. After this, matrigel was allowed to polymerize (1 hour at 37 °C). Then, 10,000 pooled HUVECs (passage 4–6, Cell Applications, Cat N°: 200pK-05n) were seeded in 50 µL of CM and cultured as usual. Time-lapse images were obtained each hour during a time period of 8 hours. Angiogenesis was assessed on the basis of formation of capillary-like structures. The number of meshes was quantified using the free angiogenesis analyzer macro from ImageJ. For each condition, three technical replicates were seeded and five independent experiments (n = 5) with CM coming from 5 different hMSCs donors were carried out.

For scratch assays, hMSCs at 100,000 cells/well were seeded in 6-well plates. After reaching confluence, cells were serum starved during 24 hours, and subsequently the cell monolayer was scratched vertically with a pipette tip. Detached hMSCs were removed with PBS and remaining cells were observed for 24 hours. Images were taken every hour. For each condition, three technical replicates were seeded, and trials with CM from hMSCs coming from 4 different donors (n = 4) were performed.

### Differentiation of hMSCs under secretome

For osteogenesis induction, hMSCs coming from 4 different donors were seeded in quadruplicates in basal medium at a cell density of 3,000 cells/cm^2^ in 96-well plates, and the following day, basal medium was replaced with osteogenesis induction medium: basal medium plus 10 mM β-glicerophosphate (Sigma, Cat N°: G9422), 0.2 mM ascorbic acid (Sigma, Cat N°: A4403) and 10 nM dexamethasone (Sigma, Cat N°: D4902). For conditions of osteogenesis in the presence of the secretome, 2X-osteogenesis induction medium and the CM were mixed at a 1:1 ratio. The CM from two independent hMSCs donors were used in these assays.

For adipogenesis differentiation in the presence of the secretome, confluent hMSCs coming from two independent donors (n = 2) were treated with adipogenesis differentiation medium at 2X concentration, by doubling the concentration of differentiation ingredients: 2 µM dexamethasone, 1 mM 3-isobutyl-1-methylxantine, and 400 µM indomethacin. The secretome was mixed with this 2X adipogenesis differentiation medium at a ratio of 1:1. The CM from 2 independent hMSCs donors were used in these assays.

### Alkaline Phosphatase activity

After 6 days of osteogenic differentiation, ALP activity was determined using 1-Step PNPP Substrate Solution (Thermo Fisher Scientific; Cat N°: 37621) following manufacturer’s instructions. For ALP data normalization, cell number was determined by CCK-8 kit (Bimake, Cat N°: B34304) in a parallel 96-well plate.

### Bodipy staining

At the end of the differentiation period (21 days of cell culture), the percentage of adipogenic differentiation was assessed by examining the presence of lipid droplets accumulation inside the cells. For this, BODIPY 493/503 (Invitrogen; Cat N°: D3922) was used to stain lipid droplets following the manufacturer’s instructions. The total adipocyte number was quantified based on BODIPY staining using ImageJ. The total cell number to obtain the percentage of adipocytes was calculated by counting DAPI staining of nuclei.

### siRNA transfection

Transient transfection assays were performed using specific commercially available siRNA for the inhibition of IGFBP7 (ON-TARGET plus SMART pool IGFBP7 siRNA, Cat N°: L-008675-00-0005) along with a non-targeting siRNA (ON-TARGET plus Non Targeting Control pool siRNA, D-001810-10-05), both from Dharmacon. The siRNA transfections were performed with 50 nM siRNA using the Dharmafect Transfection Reagent 1 for 24 hours according to the manufacturer’s intructions. To ensure IGFBP7 inhibition during the 6 days of osteogenic differentiation, another round of siRNA was performed, 72 hours after the first round.

### RNA isolation, reverse transcription and Q-RT-PCR

Total RNA from each sample was isolated with High Pure RNA Isolation kit (Roche; Cat N°: 11828665001) and was reverse transcribed using the Superscript III First Strand cDNA KIT (Thermo Fisher; Cat N°: 10308632) according to the manufacturer’s instructions. All reactions were carried out in triplicate on an AriaMX Real Time PCR System (Agilent) using Brilliant III Ultrafast SYBR Green Master mix (Agilent, Cat N°: 600882). For gene expression normalization *GAPDH* was used. Primer sequences are available upon request.

### Statistical analysis

All replicates in this study were independent biological replicates, which came from different biological samples (different hMSCs-lines donors). Unless otherwise stated, to determine statistical significance, data are derived from at least four different biological replicates and expressed as the mean ± standard deviation. Results for each individual biological replicates are based on technical replicates: triplicates in the case of angiogenesis, wound healing, adipogenesis and RT-Q-PCR and quadruplicates in the case of alkaline phosphatase activity.

The Mann-Whitney U test was used to determine statistically significant differences and p < 0.05 was considered to be statistically significant.

## Electronic supplementary material

supplementary information
