# Supplementary material for: Secretome analysis of in vitro aged human mesenchymal stem cells reveals IGFBP7 as a putative factor for promoting osteogenesis
Source: Sci Rep. 2018 Mar 15;8:4632. doi: 10.1038/s41598-018-22855-z (PMC5854613; doi:10.1038/s41598-018-22855-z)
Supplement: Supplementary file 1 — supplementary information [file 41598_2018_22855_MOESM1_ESM.pdf]

Secretome analysis of in vitro aged human mesenchymal stem cells reveals IGFBP7 as a putative factor for promoting osteogenesis

Arantza Infante* and Clara I. Rodríguez*[1]

*Stem Cells and Cell Therapy Laboratory, BioCruces Health Research Institute, Cruces University Hospital, Barakaldo 48903, Spain

[1]Corresponding author: Clara I. Rodríguez

E-mail: cirodriguez@osakidetza.eus

Supplementary information

Supplementary Table S1. Differentially secreted proteins in preA-hMSCs-CM compared *versus* ctrl-hMSCs-CM (Fold change). [1]Ab: Secreted factors detected by antibody arrays; MS: Secreted factors detected by LS-MS, [2] Role in bone metabolism; [3] Regulated secretion in osteogenesis of hMSCs; [4] Dysregulated secretion in preA-adipocytes, fold induction is indicated in parentheses, *TGFB pathway, Bold: detected by both antibody arrays and LS-MS

Protein description	Name	Fold	1	2	3	4
S100 Calcium-Binding Protein A6 (Calcyclin)	S100A6	42.82	Ab	√	√	√ (11.9)
Fibronectin	FN1*	30.72	Ab	√	√	√ (18.2)
Vascular Endothelial Growth Factor A	VEGF	29.02	Ab	√		
Transforming Growth Factor Beta Induced	TGFBI*	19.85	Ab	√	√	√ (50.1)
Transmembrane Protein With EGF Like And Two Follistatin Like Domains 2	TMEFF2	15.77	Ab			
Parathyroid Hormone Like Hormone	PTHLH	12.52	Ab	√		
Insulin Like Growth Factor Binding Protein 7	IGFBP7*	12.50	Ab	√	√	√ (3.5)
Serpin Family E Member 1	SERPINE1*	9.87	Ab	√	√	√ (2.1)
C-Type Lectin Domain Family 3 Member B	CLEC3B	9.23	Ab	√		√ (3.0)
Inhibin Beta A Subunit	INHBA*	5.82	Ab			
Fibrinogen Alpha Chain	FGA	4.84	Ab			√ (2.0)
**Pentraxin 3**	**PTX3**	4.67	Ab	√	√	
Secreted Frizzled Related Protein 4	SFRP4	4.31	Ab	√	√	√ (3.9)
Transcription Factor SOX-2	SOX2	4.31	Ab			
Fibroblast Growth Factor 7	FGF7	3.88	Ab			
Amyloid Beta Precursor Protein	APP	3.54	Ab	√	√	√ (3.1)
Thrombospondin 1	THBS1*	3.45	Ab	√	√	√ (21.7)
ADAM Metallopeptidase With Thrombospondin Type 1 Motif 10	ADAMTS10	3.14	Ab			√ (4.4)
Integrin Binding Sialoprotein	IBSP	2.85	Ab	√		√ (5.4)
Granulin	GRN	2.66	Ab	√	√	√ (2.2)
ROS Proto-Oncogene 1, Receptor Tyrosine Kinase	ROS1	2.61	Ab			√ (2.1)
Cystatin B	CSTB	2.55	Ab	√		√ (6.6)
KRAS Proto-Oncogene, GTPase	KRAS	2.43	Ab			
Legumain	LGMN	2.39	Ab	√	√	√ (3.2)
Dickkopf WNT Signaling Pathway Inhibitor 1	DKK1	2.37	Ab	√		√ (2.6)
Secreted Protein Acidic And Cysteine Rich	SPARC	2.24	Ab	√	√	
Ectodysplasin A	EDA	2.22	Ab	√		
Insulin Like Gowth Factor Binding Protein 1	IGFBP1	2.21	Ab	√		
S100 Calcium Binding Protein G	S100G	2.13	Ab			
Cathepsin B	CTSB	2.10	Ab	√	√	√ (3.3)
Calcitonin Related Polypeptide Alpha	CALCA	2.09	Ab			√ (2.4)
**Calreticulin**	**CALR**	2.08	Ab	√	√	
Solute Carrier Family 2 Member 2	SLC2A2	2.01	Ab			
Kallikrein 5	KLK5	2.01	Ab			√ (4.2)
Toll Like Receptor 3	TLR3	0.49	Ab			
Synuclein Gamma	SNCG	0.47	Ab			√ (2.6)
Matrix Metallopeptidase 24	MMP24	0.34	Ab			
Angiotensin I Converting Enzyme 2	ACE2	0.30	Ab			
Beta-2-Microglobulin	B2M	0.29	Ab	√	√	
Lymphotoxin Beta	LTB	0.21	Ab			
Interleukin 11	IL11	0.19	Ab	√		√ (2.6)
Thyroid Peroxidase	TPO	0.15	Ab			
FK506 Binding Protein 9	FKBP9	20.52	MS	√	√	
Ribosomal protein S12	RPS12	17.06	MS	√	√	
Potassium Channel Tetramerization Domain Containing 12	KCTD12	13.65	MS			
FK506 Binding Protein 10	FKBP10	13.57	MS	√		
Prosaposin	PSAP	11.49	MS	√	√	√ (0.44)
AHNAK Nucleoprotein	AHNAK	7.70	MS			
Reticulocalbin 1	RCN1	7.67	MS			
Cathepsin D	CTSD	6.70	MS	√	√	

Lectin, Mannose Binding 2	LMAN2	6.27	MS			
Nucleobindin 2	NUCB2	5.27	MS	√		
Procollagen-Lysine,2-Oxoglutarate 5-Dioxygenase 1	PLOD1	5.10	MS	√	√	
Protein Kinase C Substrate 80K-H	PRKCSH	4.47	MS			
Calumenin	CALU	4.33	MS	√	√	
Lectin, Mannose Binding 2	LMAN2	6.27	MS			
Nucleobindin 2	NUCB2	5.27	MS	√		
Procollagen-Lysine,2-Oxoglutarate 5-Dioxygenase 1	PLOD1	5.10	MS	√	√	
Protein Kinase C Substrate 80K-H	PRKCSH	4.47	MS			
Calumenin	CALU	4.33	MS	√	√	
Heat Shock Protein Family A (Hsp70) Member 5	HSPA5	4.27	MS			
Reticulocalbin 3	RCN3	3.82	MS	√	√	
Serpin Family H Member 1	SERPINH1*	3.31	MS	√	√	
Prolyl 4-Hydroxylase Subunit Beta	P4HB	3.23	MS	√	√	
Protein Disulfide Isomerase Family A Member 3	PDIA3	3.21	MS	√	√	
LIM And SH3 Protein 1	LASP1*****	3.07	MS			
Hemoglobin Subunit Alpha 1	HBA1	2.84	MS			
Annexin A2	ANXA2	2.61	MS	√	√	
**Pentraxin 3**	**PTX3**	2.61	MS	√	√	
Galectin 1	LGALS1	2.56	MS			√ (10.4)
Collagen Type VI Alpha 3 Chain	COL6A3	2.56	MS	√	√	
**Calreticulin**	**CALR**	2.40	MS	√	√	
Lactate Dehydrogenase A	LDHA	2.10	MS	√	√	√ (2.0)
Transgelin	TAGLN*	2.04	MS	√	√	
Actin Beta	ACTB	1.83	MS			
Stanniocalcin 2	STC2	1.68	MS	√	√	
Fibulin	FBLN1*	1.61	MS	√	√	
Insulin Like Growth Factor Binding Protein 3	IGFBP3	1.57	MS	√		√ (2.2)
HtrA Serine Peptidase 1	HTRA*	1.55	MS	√	√	
Procollagen C-Endopeptidase Enhancer	PCOLCE	1.48	MS	√	√	
Biglycan	BGN*	0.64	MS	√	√	
Versican	VCAN	0.54	MS			
Collagen Type I Alpha 1 Chain	COL1A1*	0.54	MS	√	√	
NPC Intracellular Cholesterol Transporter 2	NPC2	0.49	MS	√	√	
EGF Containing Fibulin Like Extracellular Matrix Protein 2	EFEMP2	0.45	MS	√	√	
Lumican	LUM*	0.43	MS	√	√	
Collagen Type IV Alpha 2 Chain	COL4A2*	0.43	MS	√	√	
Insulin Like Growth Factor Binding Protein 4	IGFBP4	0.36	MS	√	√	
Immunoglobulin Superfamily Containing Leucine Rich Repeat	ISLR	0.30	MS			
Collagen Type I Alpha 2 Chain	COL1A2*	0.30	MS	√	√	
Serglycin	SRGN	0.30	MS	√	√	√ (0.49)

Supplementary Table S2. Differentially secreted proteins in preA-adipocytes-CM compared *versus* ctrl-adipocytes-CM (Fold change). [1]Ab: Secreted factors detected by antibody arrays; MS: Secreted factors detected by LS-MS, [2] Role in bone metabolism.

**Protein description**	**Name**	**Fold**	**1**	**2**
Transforming Growth Factor Beta Induced	TGFBI	50.1	Ab	√
Thrombospondin 1	THBS1	21.7	Ab	√
Fibronectin	FN1	18.2	Ab	√
Parathyroid Hormone Like Hormone	PTHLH	17.6	Ab	
C-X-C Motif Chemokine Ligand 1	CXCL1	15.1	Ab	
Serpin Family A Member 5	SERPINA5	13.9	Ab	
Plasminogen	PLG	12.7	Ab	
Cyclin Dependent Kinase Inhibitor 1A	CDKN1A	12.6	Ab	
S100 Calcium Binding Protein A6	S100A6	11.9	Ab	√
Galectin 1	LGALS1	10.4	Ab	√
TNF Receptor Superfamily Member 4	TNFRSF4	10.1	Ab	
Leucine Rich Alpha-2-Glycoprotein 1	LRG1	10.0	Ab	
Galectin 3	LGALS3	10.0	Ab	√
Pim-2 Proto-Oncogene, Serine/Threonine Kinase	PIM2	9.5	Ab	
Diablo IAP-Binding Mitochondrial Protein	DIABLO	8.6	Ab	
Dickkopf WNT Signaling Pathway Inhibitor 3	DKK3	8.5	Ab	√
Interleukin 9	IL9	8.1	Ab	
Intercellular Adhesion Molecule 3	ICAM3	8.0	Ab	
Angiopoietin Like 4	ANGPTL4	7.5	Ab	
Chordin Like 1	CHRDL1	7.5	Ab	
Fibroblast Growth Factor 17	FGF17	6.8	Ab	
MET Proto-Oncogene, Receptor Tyrosine Kinase	MET	6.6	Ab	
Cystatin B	CSTB	6.6	Ab	
SMAD Family Member 2	SMAD2	6.3	Ab	
Interleukin 5	IL5	6.1	Ab	
Myostatin	MSTN	6.0	Ab	
Fibrinogen Beta Chain	FGB	6.0	Ab	
Syndecan 3	SDC3	5.7	Ab	
Vascular Endothelial Growth Factor A	VEGFA	5.6	Ab	
Integrin Binding Sialoprotein	IBSP	5.4	Ab	
Growth Differentiation Factor 11	GDF11	5.1	Ab	
HtrA Serine Peptidase 2	HTRA2	5.1	Ab	
Serpin Family A Member 4	SERPINA4	5.0	Ab	
Cathepsin L	CTSL	5.0	Ab	√
Colony Stimulating Factor 2 Receptor Beta Common Subunit	CSF2RB	5.0	Ab	
Interleukin 6	IL6	5.0	Ab	

C-X-C Motif Chemokine Ligand 12	CXCL12	4.9	Ab	
Growth Differentiation Factor 9	GDF9	4.9	Ab	
C-X-C Motif Chemokine Ligand 8	CXCL8	4.8	Ab	
HRas Proto-Oncogene, GTPase	HRAS	4.5	Ab	
Progesterone Receptor	PGR	4.5	Ab	
ADAM Metallopeptidase With Thrombospondin Type 1 Motif 10	ADAMTS10	4.4	Ab	
Granzyme A	GZMA	4.4	Ab	
Growth Differentiation Factor 1	GDF1	4.4	Ab	
Fibroblast Growth Factor 18	FGF18	4.3	Ab	
Interleukin 21	IL21	4.3	Ab	
Interleukin 18 Receptor 1	IL18R1	4.2	Ab	
Kallikrein Related Peptidase 5	KLK5	4.2	Ab	
Heat Shock Protein Family A (Hsp70) Member 4	HSPA4	4.2	Ab	
Endothelial Cell Adhesion Molecule	ESAM	4.1	Ab	
Tumor Necrosis Factor Superfamily Member 4	TNFSF4	4.1	Ab	
Serpin Family A Member 12	SERPINA12	4.1	Ab	
Clusterin	CLU	4.0	Ab	√
Secreted Frizzled Related Protein 4	SFRP4	3.9	Ab	√
Pancreatic And Duodenal Homeobox 1	PDX1	3.9	Ab	
Receptor-Like Tyrosine Kinase	RYK	3.9	Ab	
BMP Binding Endothelial Regulator	BMPER	3.8	Ab	
Adiponectin, C1Q And Collagen Domain Containing	ADIPOQ	3.8	Ab	
Heat Shock Protein Family B (Small) Member 6	HSPB6	3.8	Ab	
Interleukin 7	IL7	3.8	Ab	
Reversion Inducing Cysteine Rich Protein With Kazal Motifs	RECK	3.7	Ab	
Heparin Binding EGF Like Growth Factor	HBEGF	3.6	Ab	
ADAM Metallopeptidase Domain 17	ADAM17	3.6	Ab	
C-C Motif Chemokine Ligand 28	CCL28	3.6	Ab	
Gremlin 1, DAN Family BMP Antagonist	GREM1	3.6	Ab	
Interferon Gamma	IFNG	3.5	Ab	
Insulin Like Growth Factor Binding Protein 7	IGFBP7	3.5	Ab	√
Interleukin 13	IL13	3.5	Ab	
Decorin	DCN	3.5	Ab	√
Interleukin 15	IL15	3.5	Ab	
Interleukin 18 Binding Protein	IL18BP	3.4	Ab	
Cathepsin B	CTSB	3.3	Ab	√
TATA-Box Binding Protein Associated Factor 4	TAF4	3.3	Ab	
TNF Receptor Superfamily Member 8	TNFRSF8	3.3	Ab	
Frizzled Class Receptor 6	FZD6	3.3	Ab	
Presenilin 2	PSEN2	3.3	Ab	
Endothelin 1	EDN1	3.2	Ab	
Legumain	LGMN	3.2	Ab	√
Insulin Like Growth Factor Binding Protein 2	**IGFBP2**	3.1	Ab	√
Amyloid Beta Precursor Protein	APP	3.1	Ab	√
Midkine (Neurite Growth-Promoting Factor 2)	MDK	3.1	Ab	
Fas Associated Via Death Domain	FADD	3.1	Ab	
Interleukin 1 Receptor Accessory Protein	IL1RAP	3.0	Ab	
C-Type Lectin Domain Family 3 Member B	CLEC3B	3.0	Ab	
Colony Stimulating Factor 3	CSF3	3.0	Ab	
Interleukin 2	IL2	3.0	Ab	
S100 Calcium Binding Protein A12	S100A12	3.0	Ab	
Tumor Protein P53	TP53	2.9	Ab	
Chemerin Chemokine-Like Receptor 1	CMKLR1	2.9	Ab	
Frizzled Class Receptor 1	FZD1	2.9	Ab	
Tec Protein Tyrosine Kinase	TEC	2.9	Ab	

Fibroblast Growth Factor 13	FGF13	2.9	Ab	
C-X-C Motif Chemokine Ligand 14	CXCL14	2.9	Ab	
Dickkopf WNT Signaling Pathway Inhibitor 4	DKK4	2.9	Ab	
Basal Cell Adhesion Molecule (Lutheran Blood Group)	BCAM	2.9	Ab	
Interleukin 19	IL19	2.8	Ab	
Interleukin 17 Receptor D	IL17RD	2.8	Ab	
Fatty Acid Binding Protein 4	FABP4	2.8	Ab	
Semaphorin 3A	SEMA3A	2.8	Ab	
X-Linked Inhibitor Of Apoptosis	XIAP	2.8	Ab	
Proopiomelanocortin	POMC	2.8	Ab	
Fibroblast Growth Factor 2	FGF2	2.8	Ab	
Paraoxonase 2	PON2	2.8	Ab	
Serine/Threonine-Protein Kinase PAK 7	PAK7	2.8	Ab	
C-X-C Motif Chemokine Ligand 9	CXCL9	2.7	Ab	
Complement Factor D	CFD	2.7	Ab	
Glypican 5	GPC5	2.7	Ab	
Interleukin 34	IL34	2.7	Ab	
Interleukin 1 Receptor Accessory Protein Like 1	IL1RAPL1	2.7	Ab	
Endoglin	ENG	2.7	Ab	
C-C Motif Chemokine Ligand 11	CCL11	2.7	Ab	
Receptor Tyrosine Kinase Like Orphan Receptor 2	ROR2	2.7	Ab	
WNT Inhibitory Factor 1	WIF1	2.7	Ab	
Growth Differentiation Factor 3	GDF3	2.7	Ab	
C-C Motif Chemokine Ligand 2	CCL2	2.6	Ab	
Endothelial Cell Specific Molecule 1	ESM1	2.6	Ab	
Transferrin Receptor	TFRC	2.6	Ab	
Neuronal Pentraxin Receptor	NPTXR	2.6	Ab	
Thymic Stromal Lymphopoietin	TSLP	2.6	Ab	
C-C Motif Chemokine Ligand 27	CCL27	2.6	Ab	
Lymphatic Vessel Endothelial Hyaluronan Receptor 1	LYVE1	2.6	Ab	
SERTA Domain Containing 2	SERTAD2	2.6	Ab	
Macrophage Migration Inhibitory Factor (Glycosylation-Inhibiting Factor)	MIF	2.6	Ab	√
Synuclein Gamma	SNCG	2.6	Ab	
Interleukin 11	IL11	2.6	Ab	
Rho Associated Coiled-Coil Containing Protein Kinase 1	ROCK1	2.6	Ab	
Serpin Family A Member 3	SERPINA3	2.6	Ab	
Dickkopf WNT Signaling Pathway Inhibitor 1	DKK1	2.6	Ab	
Fms Related Tyrosine Kinase 1	FLT1	2.5	Ab	
Pyruvate Kinase, Muscle	PKM	2.5	Ab	
Trefoil Factor 1	TFF1	2.5	Ab	
Serpin Family B Member 5	SERPINB5	2.5	Ab	
Interferon Lambda 1	IFNL1	2.5	Ab	
C-X-C Motif Chemokine Ligand 5	CXCL5	2.5	Ab	
Frizzled Class Receptor 4	FZD4	2.5	Ab	
Chordin Like 2	CHRDL2	2.5	Ab	
CD1a Molecule	CD1A	2.5	Ab	
TNF Receptor Superfamily Member 25	TNFRSF25	2.5	Ab	
Fucosyltransferase 4	FUT4	2.5	Ab	
Geminin, DNA Replication Inhibitor	GMNN	2.5	Ab	
Family With Sequence Similarity 3 Member B	FAM3B	2.5	Ab	
Interleukin 16	IL16	2.5	Ab	
Fibroblast Growth Factor Binding Protein 1	FGFBP1	2.5	Ab	
Aminoacyl TRNA Synthetase Complex Interacting Multifunctional Protein	AIMP1	2.5	Ab	
IL2 Inducible T-Cell Kinase	ITK	2.5	Ab	

Interleukin 15 Receptor Subunit Alpha	IL15RA	2.4	Ab	
Lactotransferrin	LTF	2.4	Ab	
Matrix Metallopeptidase 11	MMP11	2.4	Ab	
Calcitonin Related Polypeptide Alpha	CALCA	2.4	Ab	
Epidermal Growth Factor	EGF	2.4	Ab	
Fas Ligand	FASLG	2.4	Ab	
Cyclin Dependent Kinase Inhibitor 1B	CDKN1B	2.4	Ab	
Transferrin	TF	2.4	Ab	
Interleukin 1 Alpha	IL1A	2.4	Ab	
C-X-C Motif Chemokine Ligand 13	CXCL13	2.4	Ab	
Fas Cell Surface Death Receptor	FAS	2.4	Ab	
Cytotoxic T-Lymphocyte Associated Protein 4	CTLA4	2.3	Ab	
Cerberus 1, DAN Family BMP Antagonist	CER1	2.3	Ab	
Protein S (Alpha)	PROS1	2.3	Ab	√
Fibroblast Growth Factor 16	FGF16	2.3	Ab	
Growth Differentiation Factor 5	GDF5	2.3	Ab	
Glutathione Peroxidase 1	GPX1	2.3	Ab	
Chitinase 3 Like 1	CHI3L1	2.3	Ab	
Galectin 3 Binding Protein	LGALS3BP	2.3	Ab	√
Hepatitis A Virus Cellular Receptor 1	HAVCR1	2.3	Ab	
APC, WNT Signaling Pathway Regulator	APC	2.3	Ab	
FER Tyrosine Kinase	FER	2.2	Ab	
RELA Proto-Oncogene, NF-KB Subunit	RELA	2.2	Ab	
C-X-C Motif Chemokine Ligand 2	CXCL2	2.2	Ab	
SPARC Like 1	SPARCL1	2.2	Ab	
Apelin Receptor	APLNR	2.2	Ab	
Insulin Like Growth Factor Binding Protein 4	IGFBP4	2.2	Ab	√
Insulin Like Growth Factor Binding Protein 3	IGFBP3	2.2	Ab	
Cardiotrophin 1	CTF1	2.2	Ab	
Tyrosine Kinase Non Receptor 1	TNK1	2.2	Ab	
CD40 Molecule	CD40	2.2	Ab	
Neurotrophic Receptor Tyrosine Kinase 2	NTRK2	2.2	Ab	
Neurofibromin 1	NF1	2.2	Ab	
Granulin Precursor	GRN	2.2	Ab	√
LDL Receptor Related Protein 6	LRP6	2.2	Ab	
Intelectin 1	ITLN1	2.2	Ab	
LYN Proto-Oncogene, Src Family Tyrosine Kinase	LYN	2.2	Ab	
C-X-C Motif Chemokine Ligand 16	CXCL16	2.2	Ab	
Ectonucleotide Pyrophosphatase/Phosphodiesterase 2	ENPP2	2.2	Ab	√
Protein Tyrosine Phosphatase, Receptor Type D	PTPRD	2.2	Ab	
Neuregulin 1	NRG1	2.2	Ab	
Insulin Like Growth Factor 2 Receptor	IGF2R	2.2	Ab	
Secreted Phosphoprotein 1	SPP1	2.1	Ab	
TIMP Metallopeptidase Inhibitor 3	TIMP3	2.1	Ab	
Interleukin 12 Receptor Subunit Beta 2	IL12RB2	2.1	Ab	
Bruton Tyrosine Kinase	BTK	2.1	Ab	
Interleukin 36, Beta	IL36B	2.1	Ab	
Troponin I3, Cardiac Type	TNNI3	2.1	Ab	
C-X3-C Motif Chemokine Ligand 1	CX3CL1	2.1	Ab	
Apolipoprotein A4	APOA4	2.1	Ab	
C-Src Tyrosine Kinase	CSK	2.1	Ab	
Muscle Associated Receptor Tyrosine Kinase	MUSK	2.1	Ab	
Alpha-2-Macroglobulin	A2M	2.1	Ab	
Retinol Binding Protein 4	RBP4	2.1	Ab	
Insulin Like Growth Factor 1	IGF1	2.1	Ab	

C-C Motif Chemokine Ligand 21	CCL21	2.1	Ab	
Rho Associated Coiled-Coil Containing Protein Kinase 2	ROCK2	2.1	Ab	
Tumor Necrosis Factor	TNF	2.1	Ab	
Vascular Endothelial Growth Factor A	VEGFA	2.1	Ab	
Neuregulin 1	NRG1	2.1	Ab	
Interleukin 6 Receptor	IL6R	2.1	Ab	
Serpin Family E Member 1	SERPINE1	2.1	Ab	√
ROS Proto-Oncogene 1, Receptor Tyrosine Kinase	ROS1	2.1	Ab	
Somatostatin Receptor 5	SSTR5	2.1	Ab	
Interleukin 10	IL10	2.1	Ab	
Cystatin A	CSTA	2.1	Ab	
Fibroblast Growth Factor Receptor-Like 1	FGFRL1	2.0	Ab	
Angiopoietin 1	ANGPT1	2.0	Ab	
Fyn Related Src Family Tyrosine Kinase	FRK	2.0	Ab	
Somatostatin Receptor 2	SSTR2	2.0	Ab	
Angiotensinogen	AGT	2.0	Ab	√
Insulin Like Growth Factor Binding Protein 1	IGFBP1	2.0	Ab	
TXK Tyrosine Kinase	TXK	2.0	Ab	
Aldolase, Fructose-Bisphosphate C	ALDOC	2.0	Ab	√
Activin A Receptor Type 1B	ACVR1B	2.0	Ab	
Bone Morphogenetic Protein 3	BMP3	2.0	Ab	
Tumor Necrosis Factor Superfamily Member 8	TNFSF8	2.0	Ab	
Junction Adhesion Molecule Like	JAML	2.0	Ab	
Tyrosine Kinase Non Receptor 2	TNK2	2.0	Ab	
Fibrinogen Alpha Chain	FGA	2.0	Ab	
Interleukin 1 Receptor Like 2	IL1RL2	2.0	Ab	
Troponin C1, Slow Skeletal And Cardiac Type	TNNC1	2.0	Ab	
Glial Cell Derived Neurotrophic Factor	GDNF	2.0	Ab	
Growth Hormone 1	GH1	2.0	Ab	
Neuregulin 1	NRG1	2.0	Ab	
Low Density Lipoprotein Receptor	LDLR	2.0	Ab	
Uromodulin	UMOD	2.0	Ab	
C-C Motif Chemokine Ligand 23	CCL23	2.0	Ab	
HCK Proto-Oncogene, Src Family Tyrosine Kinase	HCK	2.0	Ab	
Frizzled Class Receptor 3	FZD3	2.0	Ab	
Interleukin 6 Receptor	SGP130	2.0	Ab	
Albumin	ALB	0.5	Ab	
Folate Receptor 1	FOLR1	0.5	Ab	
Haptoglobin	HP	0.4	Ab	
Alpha-2-Glycoprotein 1, Zinc-Binding	AZGP1	0.2	Ab	
Tumor Necrosis Factor Superfamily Member 11	TNFSF11	0.2	Ab	
Ribosomal Protein S18	RPS18	4.3	MS	
Ribosomal Protein L18	RPL18	4.1	MS	
Filamin A	FLNA	1.6	MS	√
T-Complex 1	TCP1	1.9	MS	
Enolase 1	ENO1	1.5	MS	√
Chaperonin Containing TCP1 Subunit 5	CCT5	2.9	MS	
Collagen Type V Alpha 1 Chain	COL5A1	0.6	MS	√
Aldolase, Fructose-Bisphosphate A	ALDOA	2.4	MS	√
Vimentin	VIM	2.8	MS	√
Inter-Alpha-Trypsin Inhibitor Heavy Chain 1	ITIH1	1.9	MS	
Serglycin	SRGN	0.5	MS	√
Transketolase	TKT	2.1	MS	√
Proteasome Subunit Alpha 8	PSMA8	1.8	MS	
Chaperonin Containing TCP1 Subunit 3	CCT3	2.7	MS	

Prosaposin	PSAP	0.4	MS	√
Transgelin 2	TAGLN2	1.7	MS	√
Ribosomal Protein S3	RPS3	3.1	MS	√
Phosphoserine Aminotransferase 1	PSAT1	2.8	MS	√
Vinculin	VCL	1.8	MS	√
Heat Shock Protein Family A (Hsp70) Member 8	HSPA8	1.8	MS	√
Transaldolase 1	TALDO1	1.8	MS	√
Gelsolin	GSN	0.4	MS	√
Lactate Dehydrogenase A	LDHA	1.9	MS	√
Fatty Acid Binding Protein 5	FABP5	2.3	MS	
Alcohol Dehydrogenase 1B (Class I), Beta Polypeptide	ADH1B	2.1	MS	
Platelet Derived Growth Factor Receptor Beta	PDGFRB	0.1	MS	
Ribosomal Protein L29	RPL29	3.3	MS	
Ribosomal Protein S5	RPS5	3.0	MS	
Aspartyl-TRNA Synthetase	DARS	3.2	MS	

Supplementary Table S3. Donor hMSCs cell lines.

BM-hMSCs (Donor)	Age	Gender
1	39	Male
2	23	Male
3	29	Female
4	20	Male
5	38	Male
6	18	Male
7	25-40	Female
8	25-40	Male
